# The Yin and Yang of Nicotine: Harmful during Development, Beneficial in Adult Patient Populations

**DOI:** 10.3389/fphar.2012.00180

**Published:** 2012-10-08

**Authors:** Danielle S. Counotte, August B. Smit, Sabine Spijker

**Affiliations:** ^1^Department of Anatomy and Neurobiology, School of Medicine, University of MarylandBaltimore, MD, USA; ^2^Department of Molecular and Cellular Neurobiology, Center for Neurogenomics and Cognitive Research, VU UniversityAmsterdam, Netherlands

**Keywords:** nicotine, developmental stages, animal model, brain development, ADHD

## Abstract

Nicotine has remarkably diverse effects on the brain. Being the main active compound in tobacco, nicotine can aversively affect brain development. However, it has the ability to act positively by restoring attentional capabilities in smokers. Here, we focus on nicotine exposure during the prenatal and adolescent developmental periods and specifically, we will review the long-lasting effects of nicotine on attention, both in humans and animal models. We discuss the reciprocal relation of the beneficial effects of nicotine, improving attention in smokers and in patients with neuropsychiatric diseases, such as schizophrenia and attention deficit/hyperactivity disorder, vs. nicotine-related attention deficits already caused during adolescence. Given the need for research on the mechanisms of nicotine’s cognitive actions, we discuss some of the recent work performed in animals.

## Introduction

Smoking is the leading preventable cause of death and disability in the USA (Novick, 2000), and nicotine, the main active compound in tobacco smoke can have health effects in very different ways. Obviously, the best known is its highly addictive property. In addition, it has various more subtle effects on the brain. Two prominent features are its effect on brain developmental and on attention. Moreover, nicotine has been shown to exert a protective effect on the display of neurodegenerative diseases. The mechanisms behind the adverse and potentially beneficial effects will need more research in the years to come. This review will highlight some of the salient features of nicotine along these lines.

With respect to development, there are two main developmental periods during which individuals are at risk to come into contact with biologically relevant doses of nicotine. The first window of vulnerability is during prenatal development, when women might smoke during pregnancy. The second is the developmental period of adolescence, during which when most smokers start their habit (Chassin et al., 1996). In adulthood the effects of nicotine may be less on neuronal development, but become apparent in its acute effects on neuronal circuitry properties (for review Poorthuis et al., 2009). This has immediate consequences for the attention state of the brain (for review Counotte et al., 2011b). Related to this, smokers use nicotine to self-medicate their attention deficits (Lerman et al., 2001), and patients with attention deficit/hyperactivity disorder (ADHD) perform worse when abstinent from nicotine (McClernon et al., 2008). ADHD is a common clinically significant condition in school-aged children, affecting 5–10% of children worldwide, with persisting lifelong features (Pediatrics, 2000). There are reciprocal links between smoking and ADHD, with on one hand nicotine exposure during development increasing the risk for ADHD, and on the other hand high rates of ADHD patients that are smokers, probably (at least partly) to alleviate their attention deficits. Thus, despite negative effects of nicotine on brain development and its persistent addictive properties, there are instances in which nicotine exposure can be beneficial for an individual.

Another interesting feature of nicotine is its reported long-term benefit in protection to neurodegenerative disease (Bordia et al., 2008; Echeverria et al., 2010, for review, see Shimohama, 2009). Both nicotine and its breakdown product cotinine have been suggested as cognitive enhancers for Parkinson’s and Alzheimer’s disease in preclinical models (Bordia et al., 2008; Echeverria et al., 2010). Although potentially of interest, this aspect of nicotine action will not be discussed further here.

In this review, we will examine the long-term effects of nicotine exposure during two epochs of brain development (prenatal and peri-adolescent) in the attention domain of cognition. In humans, it is difficult to separate the effects of developmental nicotine exposure from confounding factors, such as demographics and pre-existing or co-morbid psychiatric disorders, which is why we will also review studies using animal models of developmental nicotine exposure. As recent studies have established a correlation of developmental nicotine use and attention, and studies describing the mechanisms behind these effects start to emerge, we will focus on studies assessing attention performance. Recent work using animal models has enabled us to study the molecular and synaptic mechanisms underlying the long-term effects of developmental nicotine exposure.

## Prenatal Nicotine Exposure

Even though smoking by pregnant women is declining in recent years, still 13% of women reported smoking during pregnancy in 2005 (Center for Disease Control and Prevention, 2009), which might even be an underestimation due to non-disclosure (Dietz et al., 2010). In some high-risk populations, smoking rates are as high as 25% (Arria et al., 2006). Many women find it hard to quit smoking when they are pregnant (Einarson and Riordan, 2009). Recommending nicotine replacement therapy may not be beneficial (Slotkin, 2008) considering that nicotine can cross the placenta and thus will enter the fetus through the mother’s circulation. The developing fetus does not have the abilities to breakdown nicotine and its active metabolite cotinine as efficient as adults do, so nicotine and cotinine levels will buildup in the fetus (Sastry et al., 1998). A well-known more immediate consequence of maternal smoking during pregnancy is the increased risk for sudden infant death syndrome due to nicotine targeting monoamine pathways in brainstem and cardiac sympathetic innervation (Slotkin et al., 1999, 2010), and intra-uterine growth retardation resulting in reduced birth weight (Ernst et al., 2001b). Although it does not outweigh the negative effects of smoking, smoking during (late) pregnancy could protect the mother from hypertension and resulting pre-eclampsia (England and Zhang, 2007; Wikstrom et al., 2010).

### Prenatal nicotine exposure and development of ADHD

There is a substantial body of literature of both retrospective population-based studies and case-control studies suggesting that prenatal nicotine exposure is associated with an increased occurrence of ADHD (Milberger et al., 1996; Thapar et al., 2003; Schmitz et al., 2006; Biederman et al., 2009; Galera et al., 2011; Sagiv et al., 2012; for review Winzer-Serhan, 2008; Cornelius and Day, 2009). Milberger et al. (1996) found a 2.7-fold increase in ADHD associated with maternal smoking, when comparing 140 boys with ADHD to 120 control boys and their first-degree biological relatives. Galera et al. (2011) found that prenatal tobacco exposure has a risk factor of 1.41 for attention deficits and impulsivity in a longitudinal cohort of 2057 individuals who were followed from 5 months of age to 8 years. Already shortly after birth, infants exposed to tobacco smoking *in utero* showed poorer attention skills (Espy et al., 2011). In patients with ADHD, heavy maternal smoking is associated with poorer performance on the continuous performance task (CPT; Motlagh et al., 2011). However, a causal link between maternal smoking and ADHD has not been established. Using a different experimental design examining 815 families in which infants were divided into two groups, one group that was genetically related to their parents, and one that was genetically unrelated to their mothers because of fertility treatments that used donor eggs, Thapar et al. (2009) found that ADHD was only related to maternal smoking in the genetically related infants, even though confounding factors (like parental ADHD) were controlled for. In the genetically unrelated infants, maternal smoking did lead to a decreased birth weight, but was not associated with ADHD, suggesting that in traditional observational designs it is impossible to adequately control for confounding factors. This also suggests that the link between maternal smoking and ADHD might be more complicated, involving the interaction of genetic vulnerability and environmental influences including nicotine exposure.

### Prenatal nicotine and gene × environment interactions explored in animal research

A way to have better control over confounding factors and complex gene × environment interactions in humans is to use an animal model. Importantly, nicotine in rodents was found to have similar rewarding properties and cognitive effects to humans. This makes rodent nicotine research to large extent valid to in translating to human brain development, addictive properties, and attention.

Prenatal exposure to nicotine in rodents has been shown to modulate normal developmental activation of nAChRs, which is of importance for cell survival, synapse formation, and synapse maturation (for review Dwyer et al., 2009). Rats that had been previously exposed to nicotine *in utero*, in a paradigm where mothers were exposed to 0.06 mg/ml nicotine in the drinking water before and during pregnancy, show impairments in attention performance in the 5-CSRTT, both when tested during adolescence and adulthood (Schneider et al., 2011, 2012). Specifically, animals tested as adults showed decreased correct responses and an increased number of omissions (not paying attention; Schneider et al., 2011). Animals tested during adolescence only showed an increase in anticipatory responses but no difference in accuracy (Schneider et al., 2012). Also, prenatal nicotine exposure caused increased motor impulsivity in adult animals, indicated by the increased number of anticipatory responses, but it did not cause an increase in impulsive choice, since there was no difference in delay-discounting (Schneider et al., 2011). In addition, in a paradigm modeling third trimester nicotine exposure, where pups were exposed to 6 mg/kg nicotine per day by gastric intubation from P1 to P7, it was confirmed that this did not lead to differences in impulsive choice, and similarly, there were no differences in risky decision-making (Mitchell et al., 2012). Together, these findings show that prenatal nicotine exposure does lead to attention deficits, but animals show only some of the cognitive deficits (e.g., impulsive action) that are observed in humans. This suggests that some deficits may be due to human-specific genetic or environmental factors, or are due to other components than nicotine in tobacco smoke (Baker et al., 2004).

## Adolescent Nicotine Exposure

Brain development continues during adolescence, and nicotine from tobacco smoke can interfere with normal development (for review Slotkin et al., 2007; Counotte et al., 2011b), thus leading to deficits in attention and impulsivity. Jacobsen et al. (2005, 2007) showed that adolescent smokers perform worse on working memory and attention tasks. Individuals who were exposed to prenatal maternal smoking were even more severely impaired than those that were not (Jacobsen et al., 2007). Similarly, there were gender differences; female adolescent smokers were impaired on both a visual and auditory attention task, whereas male adolescent smokers were only impaired on the auditory attention task (Jacobsen et al., 2007). It is important to note that the smokers were allowed to smoke during a break between the tests to make sure they were not inattentive because of withdrawal from nicotine (West and Hack, 1991). These deficits in attention are accompanied by reduced attention-associated prefrontal cortical blood-oxygen level dependent (BOLD)-responses (Musso et al., 2007), indicating the importance of the prefrontal cortex in attention.

Smokers in general have been reported to have higher levels of impulsivity, both impulsive choice (Bickel et al., 1999; Mitchell, 1999) and impulsive action, or inhibitory control (Mitchell, 1999; Spinella, 2002; Skinner et al., 2004). However, it is difficult to determine whether nicotine exposure leads to impulsivity, or that impulsivity leads people to start smoking. There is currently no data showing that adolescent smokers have increased impulsivity or that adolescent nicotine exposure has long-lasting effects on impulsivity.

To address the issue of causality, we used an animal model to study the long-term effect of adolescent nicotine exposure on attention and impulsivity and found that 10 days of adolescent nicotine exposure (three daily injections of 0.4 mg/kg) impairs attention in the 5-CSRTT in adult animals, even after a relatively long nicotine-free period (Counotte et al., 2009, 2011a). Also, these animals showed increased motor impulsivity, indicated by an elevated number of premature responses in the 5-CSRTT but no deficits in impulsive choice (Counotte et al., 2009). We found that the decrease in attention following adolescent nicotine exposure was (at least in part) caused by a decreased synaptic expression of the metabotropic glutamate receptor mGluR2 in the medial prefrontal cortex, because stimulation of this receptor by local infusion of an mGluR2/3 agonist relieved the attention deficits and brought the adolescent nicotine exposed animals back to the level of their control counterparts (Counotte et al., 2011a). This change in mGluR2 signaling in the mPFC in turn leads to an alteration of the rules for spike timing-dependent plasticity, meaning that the ability to filter information has decreased (Goriounova and Mansvelder, 2012). Thus, adolescent nicotine exposure affects synaptic signaling mechanisms involving metabotropic glutamate signaling in the mPFC. These signaling mechanisms are known to be important for plasticity and synaptic maturation (Michalon et al., 2012).

## Beneficial Effects of Nicotine-Like Substances

Nicotine has complex effects on cognitive performance that are in part determined by the existing state of the cholinergic system and by signaling via nicotinic receptors. Smokers, healthy non-smokers, and patients with impaired prefrontal cortical function, all differ in to what extent nicotine affects their cognitive performance. In healthy subjects, nicotine has no or only weak effects on cognitive performance. However, subjects with suboptimal performance, such as patients with ADHD, schizophrenia, or Alzheimer’s disease are more likely to benefit from nicotine, and nicotinic drugs can act beneficial on attention and sensory gating (Newhouse et al., 2004). Compared with the percentage of smokers in the general population [currently around 20% in the US (Services and U. S. Department of Health and Human Services, 2010), a higher percentage of mentally ill patients smoke regularly (26–88%, depending on the mental illness; Lasser et al., 2000)]. Particularly patients with schizophrenia, depression, post-traumatic stress disorder, or ADHD smoke, and they have a lower chance of quitting smoking (Lambert and Hartsough, 1998; Services and U. S. Department of Health and Human Services, 2010). It has been postulated that tobacco smoking may ameliorate some of the major cognitive deficits in mentally ill patients and may act as self-medication (Lerman et al., 2001; Newhouse et al., 2004). On the other hand, abstinence worsens performance on attention tasks such as the human CPT in ADHD patients, but not in controls (McClernon et al., 2008). In healthy non-smokers the evidence of beneficial effects from nicotine on cognition is less clear. Performance in some cognitive tasks shows improvement by nicotine, whereas other aspects of cognition are impaired (Levin et al., 1997, 1998; Ernst et al., 2001a; Wignall and de Wit, 2011). For example, in non-smokers, nicotine can lead to faster reaction times in attention and working memory tasks, although this improvement might come at the expense of fewer correct responses (Le Houezec et al., 1994; Foulds et al., 1996). It is of note that even in smokers, the presence of nicotine may not necessarily improve their attention to the levels of their non-smoking counterparts. In a study by Jacobsen et al. (2007) adolescent smokers were allowed to smoke during a midway break on an attention task, and they still performed worse than non-smokers.

The dichotomy in the beneficial effects of nicotine and related compounds is probably due to alterations in the cholinergic and/or cortical attention system of smokers and patients with distinct psychiatric diseases. Various factors maybe causing this. For instance, it might relate to some extent to smoking-induced changes in nicotinic receptor number and sensitivity (Kadir et al., 2006; Brasic et al., 2012), or alternatively, disease-specific developmental disturbances in receptor expression or aberrant development of circuitry may underlie this.

In smokers, whose nicotinic acetylcholine receptor signaling pathways have undergone adaptations due to chronic nicotine exposure, nicotine can be beneficial for attention performance. However, this beneficial effect of nicotine is in the context of generally impaired attention and cognitive ability after nicotine deprivation (Kleykamp et al., 2011; Vossel et al., 2011). In fact, the effect of nicotine, as that of many other drugs, resembles an inverted U-shape function in which subjects who perform at suboptimum levels will show increased performance after drug stimulation (Newhouse et al., 2011). Thus, smoking or nicotine administration in nicotine-dependent smokers only reverses the impairment in cognitive function caused by abstinence from smoking (Sacco et al., 2004).

**Figure 1:**
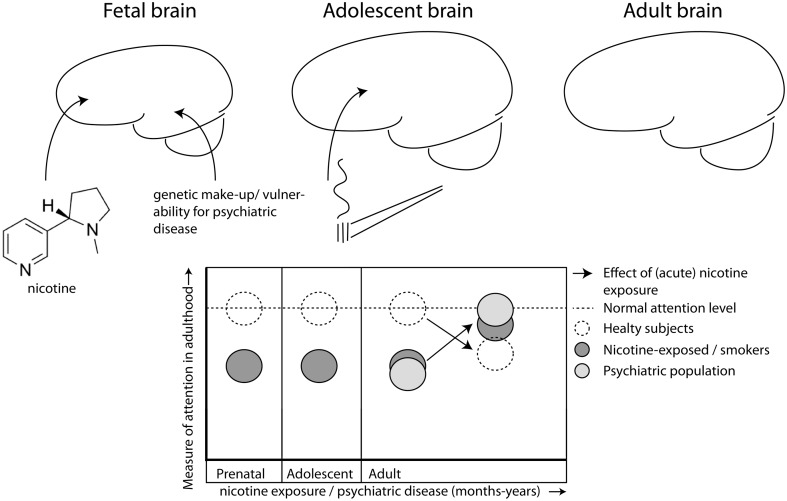
**Nicotine exposure during prenatal and/or adolescent development has long-lasting detrimental effects on attention, even after prolonged abstinence**. Exposure during both developmental periods (dark gray) has additive effects leading to worse performance than healthy controls (open, hatched line) during adulthood. Patients with certain psychiatric disorders (e.g., ADHD, schizophrenia; light gray) also suffer from decreased attention performance. Nicotine has beneficial effects on attention, but only in those individuals who have decreased levels of attention to begin with, like smokers (Lawrence et al., 2002) and patient populations (Lerman et al., 2001; Newhouse et al., 2004), but not in healthy controls (hatched line; Levin et al., 1997, 1998; Wignall and de Wit, 2011), albeit that nicotine is not always beneficial for smokers (Jacobsen et al., 2005, 2007).

This difference in altered state of nicotine sensitive pathways is reflected in studies in which compounds, directly or indirectly targeting nAChRs, are tested in (pre)clinical trials for their cognitive enhancing effects. One of these, AZD3480/TC1734, a partial agonist of α4β2* nAChR, exhibits memory-enhancing properties in healthy rodents and man (Obinu et al., 2002; Dunbar et al., 2007), albeit with mixed results in different patient populations (Dunbar et al., 2011; Frolich et al., 2011; Velligan et al., 2012). Despite initial positive reports on the effects in a phase II clinical trial, in which AZD3480 seemingly improved symptoms of ADHD, as well as results on the Stop Signal Reaction task, no further publication has yet appeared. In rats however, AZD3480 has been shown to improve MK801-induced impairments in accuracy as measured in an operant signal detection task, without having an effect on its own (Rezvani et al., 2012). Thus, novel drugs targeting the nicotinic receptors might have beneficial effects in a brain in which cholinergic signaling is disturbed (Figure 1).

## Future Perspectives

Nicotine acts in the brain via a complex repertoire of receptor subtypes. Not surprisingly, many of the precise underlying mechanisms of action of nicotine on cognitive function still need to be revealed. Experimental work on animal models might assist in this.

Results from clinical and preclinical studies show that efficacy of nicotinic receptor targeting drugs could in principle be profoundly influenced by differences in the state of the cholinergic system and/or that of the involved circuitry, and hence possibly the health or disease status of the individual. Nicotinic-compounds are promising as cognitive enhancers, and most likely act only in patients. This has the consequence that the type of animal model used to screen treatment efficacy should well fit the disease state targeted. Modeling such a state at the preclinical level can be achieved with genetic alterations, or by using pharmacological agents. Nicotine exposure during specific developmental periods maybe one of these, thereby assuring that stable alterations in receptor levels or signaling state of patients is mimicked. In addition, studying animals carrying human gene mutations offers the possibility of specifically addressing the functional role in behavioral output related to the human disease (Trueman et al., 2012). Moreover, it should be noted that “cognitive improvement” is a broad concept, and that preclinical models should try to address a large panel of behavioral phenotypes, including altered states of attentional performance, which are in general more difficult to address. Current improvements in technology and animal behavioral paradigms hold a promise for the further mechanistic understanding of the effects of nicotine on the brain (Endo et al., 2011; Winter and Schaefers, 2011).

## Conflict of Interest Statement

The authors declare that the research was conducted in the absence of any commercial or financial relationships that could be construed as a potential conflict of interest.
